# Kelch 13-propeller polymorphisms in *Plasmodium falciparum* from Jazan region, southwest Saudi Arabia

**DOI:** 10.1186/s12936-020-03467-3

**Published:** 2020-11-10

**Authors:** Ommer Mohammed Dafalla, Mohammed Alzahrani, Ahmed Sahli, Mohammed Abdulla Al Helal, Mohammad Mohammad Alhazmi, Elsiddig Mohammed Noureldin, Waheed Sideeg Mohamed, Tajeldin Bashir Hamid, Aymen Awad Abdelhaleem, Yahya Ali Hobani, Ommar Ali Arif, Ibrahim Munagi Bokar, Abdulazai Mohammed Hakami, Zaki Manawar Eisa

**Affiliations:** 1 National Center for Diseases Prevention and Control, Jazan, Saudi Arabia; 2 Zoonotic and Vector – Borne Diseases Ministry of Health, Riyadh, Saudi Arabia; 3 Department of Medicine, King Fahad Central Hospital Ministry of Health, Jazan, Saudi Arabia; 4 Medical Research Center, Jazan University, Jazan, Saudi Arabia

**Keywords:** *Plasmodium falciparum*, *k13* polymorphism, Jazan, Saudi Arabia

## Abstract

**Background:**

Artemisinin-based combination therapy (ACT) is recommended at the initial phase for treatment of *Plasmodium falciparum*, to reduce morbidity and mortality in all countries where malaria is endemic. Polymorphism in portions of *P. falciparum* gene encoding kelch (K13)-propeller domains is associated with delayed parasite clearance after ACT. Of about 124 different non-synonymous mutations, 46 have been identified in Southeast Asia (SEA), 62 in sub-Saharan Africa (SSA) and 16 in both the regions. This is the first study designed to analyse the prevalence of polymorphism in the *P. falciparum k13*-propeller domain in the Jazan region of southwest Saudi Arabia, where malaria is endemic.

**Methods:**

One-hundred and forty *P. falciparum* samples were collected from Jazan region of southwest Saudi Arabia at three different times: 20 samples in 2011, 40 samples in 2016 and 80 samples in 2020 after the implementation of ACT. *Plasmodium falciparum kelch13* (*k13*) gene DNA was extracted, amplified, sequenced, and analysed using a basic local alignment search tool (BLAST).

**Results:**

This study obtained 51 non-synonymous (NS) mutations in three time groups, divided as follows: 6 single nucleotide polymorphisms (SNPs) ‘11.8%’ in samples collected in 2011 only, 3 (5.9%) in 2011and 2016, 5 (9.8%) in 2011 and 2020, 5 (9.8%) in 2016 only, 8 (15.7%) in 2016 and 2020, 14 (27.5%) in 2020 and 10 (19.6%) in all the groups. The BLAST revealed that the 2011 isolates were genetically closer to African isolates (53.3%) than Asian ones (46.7%). Interestingly, this proportion changed completely in 2020, to become closer to Asian isolates (81.6%) than to African ones (18.4%).

**Conclusions:**

Despite the diversity of the identified mutations in the *k13*-propeller gene, these data did not report widespread artemisinin-resistant polymorphisms in the Jazan region where these samples were collected. Such a process would be expected to increase frequencies of mutations associated with the resistance of ACT.

## Background

Malaria is considered to be one of the major public health problems caused by the *Plasmodium* species. More than 100 species of *Plasmodium* have been identified [1]. While an estimated 228 million cases of malaria occurred worldwide in 2018, World Health Organization (WHO) African Region still bears the largest burden of malaria morbidity, with 213 million cases (93%) in 2018, followed by the WHO Southeast Asia Region (3.4%) and the WHO Eastern Mediterranean Region (2.1%), with the estimated deaths due to malaria globally being 405,000 cases and more than 90% of the deaths caused by *Plasmodium falciparum* [2].

During the past decades, *P. falciparum* has demonstrated great capabilities in developing its drug resistance capacity. After generating parasites resistant to chloroquine (CQ) [3], sulfadoxine-pyrimethamine (SP) [4, 5], quinine, and mefloquine, resistance to artemisinins is now spreading in some areas where *P. falciparum* species are endemic.

National anti-malarial drugs policy in Saudi Arabia adopted artemisinin based combination therapy (ACT) in 2004. In brief, the use of CQ and SP was replaced in the national treatment guidelines with artesunate and SP as first-line treatment of uncomplicated falciparum malaria and artemether-lumefanthrine (Coartem®) for treatment failures, while CQ plus 14-day treatment using primaquine was retained for the treatment of both *Plasmodium vivax* and *Plasmodium ovale*. For severe malaria, the first-line treatment is intravenous/intramuscular artesunate, the second is intramuscular artemether and the third is intravenous quinine. The recommended prophylaxis for travellers is mefloquine, while Malarone® is recommended for military personnel.

A single dose of primaquine (0.25 mg base/kg body weight (bw), maximum dose 15 mg) should be added as a gametocytocidal medicine on the first day of ACT for uncomplicated falciparum malaria. The treatments are provided free of charge [6–8]. ACT is recommended as first-line treatment for *P. falciparum* to reduce morbidity and mortality in all countries where malaria is endemic [9]. ACT contains the peroxide group, extracted and isolated from the leaves of *Artemisia annua*. ACT drugs and related compounds play a role in killing *P. falciparum* by inhibiting the activity of phosphatidylinositol-3-kinase [10].

Artemisinin resistance is defined as delayed parasite clearance; it represents a partial resistance that has affected only ring-stage parasites thus far. Nevertheless, the majority of patients with delayed parasite clearance are still able to clear their infections, following treatment with ACT with an effective partner drug or with an artesunate treatment lasting 7 days [11].

Many researches considered that polymorphism in portions of *P. falciparum* gene encoding *kelch* (*K13*)-propeller domains is associated with delayed parasite clearance after ACT. Of about 124 different non-synonymous mutations, 46 have been identified in Southeast Asia (SEA), 62 in sub-Saharan Africa (SSA) and 16 in both the regions [12], but the WHO, depending on in vivo and in vitro assays, reported only 9 *kelch13* mutations as having been validated (*F446I, N458Y, M476I, Y493H, P533L, R539T, I543T, R561H, C580Y*), 11 *kelch13* mutations labelled as associated (*P441L, G449A, C469F, A481V, P527H, N537I, G538V, V568G, P574L, F673I, A675V)* and others reported to be associated with delayed clearance, but without statistical significance due to the low number of cases (*D452E, C469Y, K479I, R515K, S522C, N537D, R575K, M579I, D584V, P667T, H719N*) [10].

The hazard of ACT-resistant parasites scattering from western Cambodia to southwest Saudi Arabia occurred earlier with CQ and SP-resistant parasites. This is the first study designed to analyse the prevalence of polymorphism in the *P. falciparum k13*-propeller domain in the Jazan region of southwest Saudi Arabia, where malaria is endemic, and the spread of artemisinin resistance may be a serious threat for malaria control and elimination.

## Methods

### Study area

This study was carried out at Beash Governate in Jazan Region of southwest Saudi Arabia, lying between 16°–12, and 18°–25, latitude north.

### Sampling

One-hundred and forty samples were collected at three different time periods after the implementation of ACT as first-line treatment of malaria in the Jazan region in 2004. The available samples from past years are: 20 DNA samples from 2011, 40 DNA samples from 2016 and 80 samples representing more than 25% of the total local samples diagnosed positive for *P. falciparum* in 2020—to date. All samples were confirmed positive for *P. falciparum* by microscopy and confirmed by nested PCR to exclude the mixed infection.

### DNA extraction

DNA was extracted from whole blood after microscopy confirmation for *P. falciparum* using Thermo Scientific GeneJET Genomic DNA Purification Kit, following manufacture’s procedures.

### Amplification of *Plasmodium falciparum*

Nested PCR was carried out for detection and identification of *P. falciparum* species, as described by Snounou et al*.* [13]. DNA samples were amplified by oligonucleotide primers obtained from Integrated DNA Technology (Belgium). These primers target the *Plasmodium* small sub-unit ribosomal RNA (ssRNA) genes. Primer pairs rPLU5 and rPLU6 were used to detect the *Plasmodium* genus in primary amplification and species-specific primers rFAL1/rFAL2 (*P. falciparum*), and rVIV1/ rVIV2 for nested PCR reaction. Primers and PCR conditions are shown in Table 1.

**Table 1:** Oligonucleotide primers and PCR conditions used in this study

Primer name	Sequence (5′-3′)	Size (bp)	PCR condition
rPLU5 rPLU6	cctgttgttgccttaaacttc ttaaaattgttgcagttaaaacg	1100	95 °C × 3 min, 35 cycles (94 °C × 60 s, 60 °C × 90 s, 72 °C × 90 s), 72 °C × 10 min
rFAL1 rFAL2	ttaaactggtttgggaaaaccaaatatatt acacaatgaactcaatcatgactacccgtc	205	95 °C × 3 min, 35 cycles (94 °C × 60 s, 55 °C × 90 s, 72 °C × 90 s), 72 °C × 10 min
rVIV2 rVIV1	cgcttctagcttaatccacataactgatac acttccaagccgaagcaaagaaagtcctt	120	95 °C × 3 min, 35 cycles (94 °C × 60 s, 55 °C × 90 s, 72 °C × 90 s), 72 °C × 10 min
K13 PCR F K13 PCR R	gggaatctggtggtaacagc cggagtgaccaaatctggga	2097	95 °C × 10 min, 35 cycles (94 °C × 60 s, 55 °C × 90 s, 72 °C × 90 s), 72 °C × 10 min
K13 Nested F K13 Nested R	gccttgttgaaagaagcaga gccaagctgccattcatttg	849	95 °C × 5 min, 35 cycles (94 °C × 60 s, 60 °C × 90 s, 72 °C × 90 s), 72 °C × 10 min

### Amplification of *Plasmodium falciparum k13*

DNA samples were amplified by oligonucleotide primers obtained from Macrogen (South Korea). These primers are designed to genotype point mutations on chromosome 13 (PF3D7-1343700) in Kelch protein propeller domain of *P. falciparum*, as described previously [14–17]. K13 PCR forward and reverse primers are designed to amplify the region 1,724,435–1,726,531 in chromosome 13 sequence of *P. falciparum* 3D7 (GenBank Accession Number, CP017003.1), and the K13 nested primers to amplify 1,724,469–1,725,317 base pair (bp) in chromosome 13 (GenBank Accession Number, CP017003.1).

### PCR reaction

In brief, primary and nested PCR were carried out in total 50 µl reaction volume, each containing 25 µl GoTag®G2 green master mix ready to use from Promega and 25 µM of each primer. Five µl of extracted DNA was used as a sample for the primary amplification and 2 µl of PCR product for the nested PCR. In each run, negative and positive controls were included. Thermal cycling was done in T100 thermal cycler (Bio-Rad, USA). Primers and PCR conditions are shown in Table 1.

The PCR products of nested amplification were analysed by gel electrophoresis (1.5 agarose in Tris–Acetate-EDTA buffer) staining with ethidium bromide. The visualization was carried out using Gel Doc XR Imaging System (Bio-Rad).

### Sequencing and data analysis

The nested PCR products of the positive samples K13 were sent to the Macrogen Co. Ltd for sequencing as follows: sequencing reactions were performed in a MJ Research PTC-225 Peltier Thermal Cycler using ABI PRISM® BigDyeTM Terminator Cycle Sequencing Kits with AmpliTaq® DNA polymerase (FS enzyme) (Applied Biosystems), following the protocols supplied by the manufacturer. Single-pass sequencing was performed on each template using K13 Nested F primer. The fluorescent-labelled fragments were purified from the unincorporated terminators with BigDye® XTerminator™ purification protocol. The samples were re-suspended in distilled water and subjected to electrophoresis in an ABI 3730xl sequencer (Applied Biosystems). K13 mutations with high frequencies confirmed by bidirectional sequencing using the K13 Nested reverse and forward primers (Table 1).

The Basic Local Alignment Search Tool (BLAST) was used to analyse and compare nucleotide sequences from samples to the reference genome PF3D7_1343700, with all mutant samples analysed and checked individually for non-synonymous mutations in *k13* propeller.

### Results

A total of 140 clinical samples collected from suspected malaria patients in Jazan region, and after microscopy diagnosis and nested PCR confirmed *P. falciparum*, the *k13* propeller domain was amplified (848 bp) by nested PCR, sequenced and analysed successfully for *k13* polymorphisms. Samples were distributed depending on the collection date: 20 samples were collected during 2011, 40 samples during 2016 and 80 samples in 2020.

### *Pfkelch13* propeller mutations in 2011 samples

**Fig. 1:**
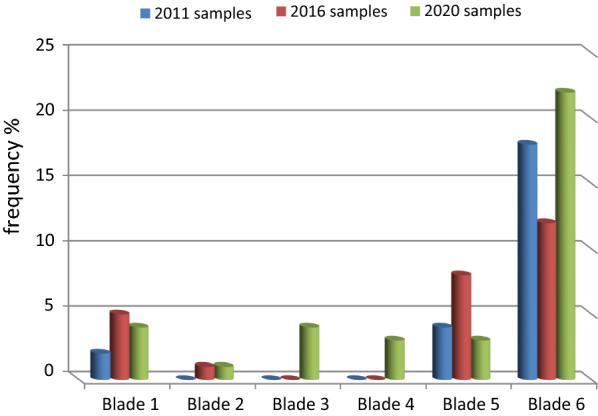
Distribution of SNPs in the *k13* propeller blades

Depending on the three case dates, the results revealed the appearance of 24 *k13* non-synonymous (NS) mutations in 2011 samples with different frequencies (Table 2). Locations of single nucleotide polymorphisms (SNPs) were distributed in blade 1 (8.3%), blade 5 (16.7%) and blade 6 (75%) (Fig. 1). Three out of 24 SNPs were reported in the SEA region in the same codon position (*P441A*, *S623N*, *F673S),* two were reported in the SSA region (*V666A*, *D680N*), and three mutations in both the regions (*F446L*, *A675S*, *A676V*). Only one SNP(*F446L)* was reported by the World Health Organization (WHO) [11] as validated mutation and two reported as associated but not validated by in vitro data (*P441A*, *F673S)* (Table 3). These mutations were present individually at low frequencies, contained different amino acids and disappeared in 2016 samples and 2020 samples.

**Table 2:** Frequencies of mutation appearance in three time groups (%)

No	Mutation	2011 samples (N = 20)	2016 samples (N = 40)	2020 samples (N = 80)	Total (N = 140)
1	*P441A	1 (5%)			1 (0.7%)
2	*P443T	–	–	2 (2.5%)	2 (1.4%)
3	***F446L	1 (5%)			1 (0.7%)
4	*C447R*			4 (5%)	4 (2.9%)
5	***F451Y***		4 (10%)		4 (2.9%)
6	**V454A		1 (2.5%)	1 (1.25%)	2 (1.4%)
7	***L457S***		4 (10%)		4 (2.9%)
8	*N458T		6 (15%)	3 (3.75%)	9 (6.4%)
9	*M476I		1 (2.5%)	1 (1.25%)	2 (1.4%)
10	***N531S***			1 (1.25%)	1 (0.7%)
11	***N537S***			1 (1.25%)	1 (0.7%)
12	*G538N			1 (1.25%)	1 (0.7%)
13	***S550C***			1 (1.25%)	1 (0.7%)
14	***P574R***			1 (1.25%)	1 (0.7%)
15	***A578G***			1 (1.25%)	1 (0.7%)
16	**D584V	–	–	4 (5%)	4 (2.9%)
17	*S623N	1 (5%)	2 (5%)	–	3 (2.1%)
18	**A626P	–	2 (5%)	–	2 (1.4%)
19	***A627P***	–	2 (5%)	1 (1.25%)	3 (2.1%)
20	***Y630F	–	2 (5%)	–	2 (1.4%)
21	***D648N***	–	4 (10%)	–	4 (2.9%)
22	***Q652P***	–	2 (5%)	1 (1.25%)	3 (2.1%)
23	***N657I***	3 (15%)	2 (5%)	1 (1.25%)	6 (4.3%)
24	***K658I***	4 (20%)	1 (2.5%)	–	5 (3.6%)
22	***L663V***	1 (5%)	3 (7.5%)	–	4 (2.9%)
26	**V666A	1 (5%)	1 (2.5%)	1(1.25%)	3 (2.1%)
27	***K670E***	3 (15%)	–	–	3 (2.1%)
28	***M671I***	2 (10%)	2 (5%)	2 (2.5%)	6 (4.3%)
29	*F673S	2 (10%)	–	–	2 (1.4%)
30	***A675S	1 (5%)	–	1 (1.25%)	2 (1.4%)
31	***A676V	1 (5%)	–	–	1 (0.7%)
32	***T677S***	3 (15%)	–	–	3 (2.1%)
33	***L678S***	3 (15%)	–	2 (1.25%)	5 (3.6%)
34	*S679A	2 (10%)	–	3 (3.75%)	5 (3.6%)
35	**D680N	1 (5%)	1 (2.5%)	1 (1.25%)	3 (2.1%)
36	***I684T***	2 (10%)	1 (2.5%)	2 (2.5%)	5 (3.6%)
37	***T685S***	2 (10%)	1 (2.5%)	2 (2.5%)	5 (3.6%)
38	***G687R***	–	–	2 (2.5%)	2 (1.4%)
39	***E688A***	3 (15%)	1 (2.5%)	2 (2.5%)	6 (4.3%)
40	***N689S***	–	–	6 (7.5%)	6 (4.3%)
41	***N689G***	–	–	5 (6.25%)	5 (3.6%)
42	***V692I***	2 (10%)	2 (5%)	4 (5%)	8 (5.7%)
43	***L693R***	–	1 (2.5%)	4 (5%)	5 (3.6%)
44	***L693G***	1 (5%)	–	4 (5%)	5 (3.6%)
45	***N694I***	–	–	2 (2.5%)	2 (1.4%)
46	***F699C***	1 (5%)	1 (2.5%)	4 (5%)	6 (3.6%)
47	***F699I***	–	1 (2.5%)	4 (5%)	5 (3.6%)
48	***P701L***	–	–	6 (7.5%)	6 (4.3%)
49	***D702H***	2 (10%)	3 (7.5%)	10 (12.5%)	15 (10.7%)
50	***T703A***	1 (5%)	–	3 (3.75%)	4 (2.9%)
51	***L708H***	–	2 (5%)	2(2.5%)	4 (2.9%)

^*^Mutations observed in SEA region,

^**^ Mutations observed in SSA region,

^***^Mutations observed in both region

Bold for the mutations detected for the first time

**Table 3:** Candidate and validated resistance mutations detected in the K13 propeller domain

Codon position	Validated	Associated	2011 samples	2016 samples	2020 samples
*P441A (0,7%)		√	√		
*F446L (0,7%)	√		√		
*N458T (6.4%)	√			√	√
**M476I (1.4%)	√			√	√
*N537S (0,7%)		√			√
*P574R (0,7%)		√			√
*F673S (1.4%)		√	√		
*A675S (0,7%)		√			√

^*^validated or associated in the same codon position but with different amino acid

^**^ validated or associated in the same codon position and amino acid

### *Pfkelch13* propeller mutations in 2016 samples

Twenty-six SNPs were identified in 2016 samples (Table 2). Mutations codon positions were located in blade 1(19.2%), blade 2 (3.8%), blade 5 (30.8%) and blade 6 (46.2%) (Fig. 1). Seven mutation codon positions have been reported before (12), two (*N458T*, *M476I*) in the SSA region, four *(V454A*, *A626P*, *V666A*, *D680N*) in the SEA region and *Y630F* in both the regions. *N458T* (different amino acids) and *M476I* were reported by the WHO [11] before as validated mutations (Table 3).

### *Pfkelch13* propeller mutations in 2020 samples

Sequencing of *k13* propeller in 80 samples collected in 2020 revealed that 37 SNPs (Table 2) were distributed in blade 1 (10.8%), blade 2 (2.7%), blade 3 (10.8%), blade 4 (8.1%), blade 5 (8.1%), and blade 6 (59.5%) (Fig. 1). Eleven of the NS mutations were described before, five of them (*P443T, N458T*, *M476I*, *G538N, P574R)* observed in the SEA region alone, four (*V454A*, *D584V, V666A*, *D680N*) in the SSA region and two (*N537S, A675S)* in both the regions. *N458T* (different amino acids) and *M476I* were reported by the WHO [11] before as validated mutations, while the codon mutations position (containing different amino acids) *P574R,* *A675S* and *N537S* considered to be associated with a parasite clearance (Table 3). Table 2, which is a cumulative summary of all SNPs with different frequencies in all the groups, also shows the distribution of these SNPs to the time groups.

Some mutations were exclusive to one group and some of them to two groups, or present in all the time groups. There were several non-synonymous mutations reported for the first time in this study.

**Fig. 2:**
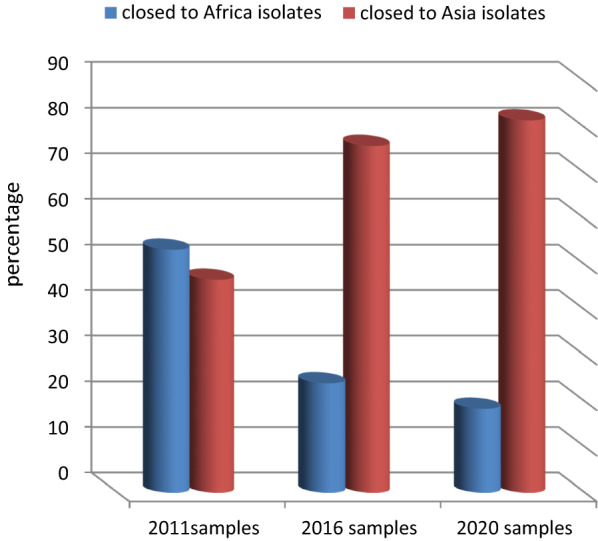
Relationship between isolates from Jazan region and isolates from African and Asian countries

**Fig. 3:**
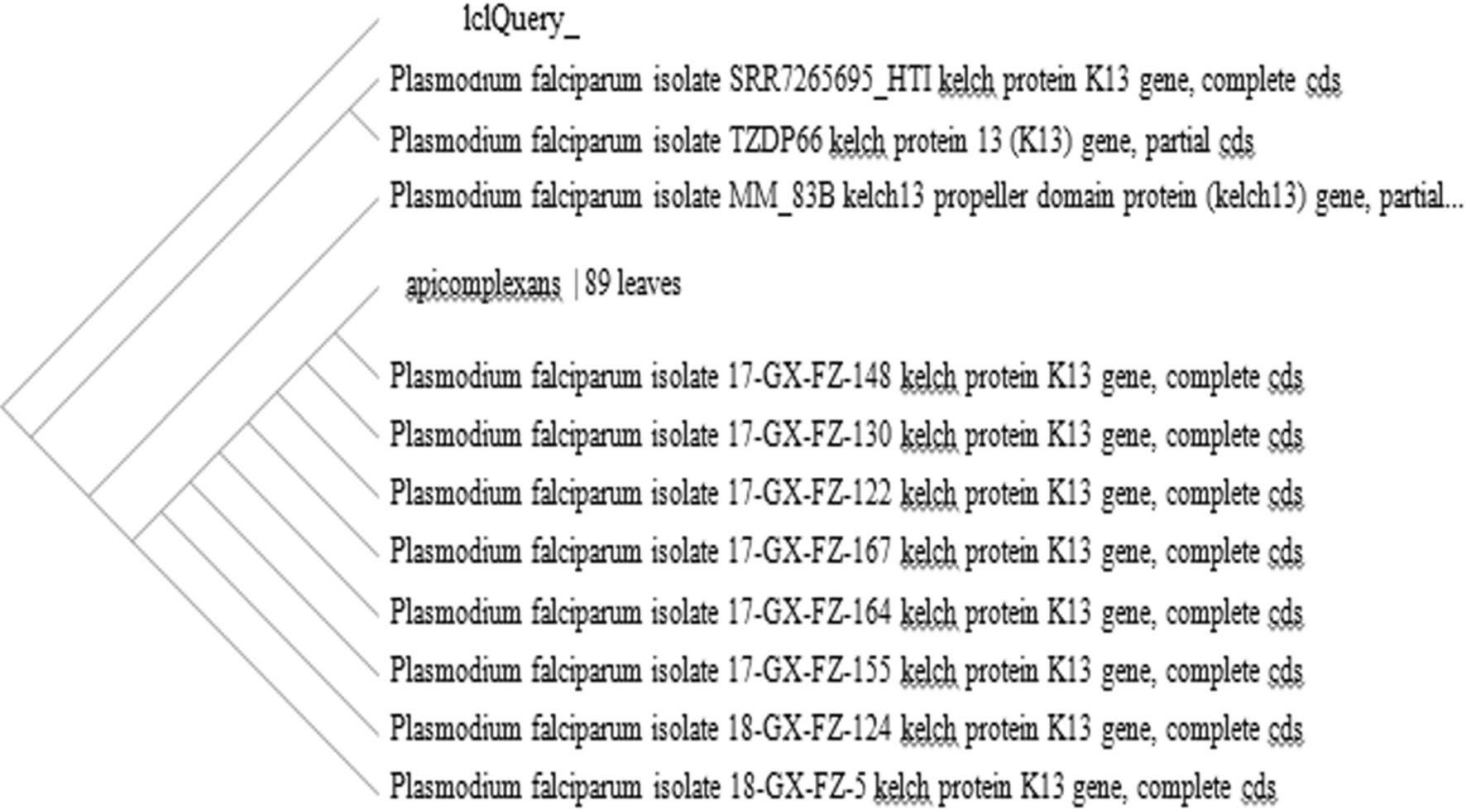
Similarity tree for one sample as an example (lclQuery) using Basic Local Alignment Search Tool program

One of the very important outcomes resulting from the BLAST analysis are strains isolated from 2011 samples, which were closely related to those isolated from African and Asian countries by 53.3% and 46.7%, respectively. While in 2020, samples were closer to African countries (24%) and closer to the Asian isolates by 76%. Notably, iIn 2020 samples, the percentage of isolated strains closer to African countries (e.g., Kenya, Nigeria, Mali) decreased to 18.4%, whereas the percentage of isolated strains closer to Asian countries (e.g., China, India, Singapore) increased to 81.6% (Figs. 2 and 3).

**Fig. 4:**
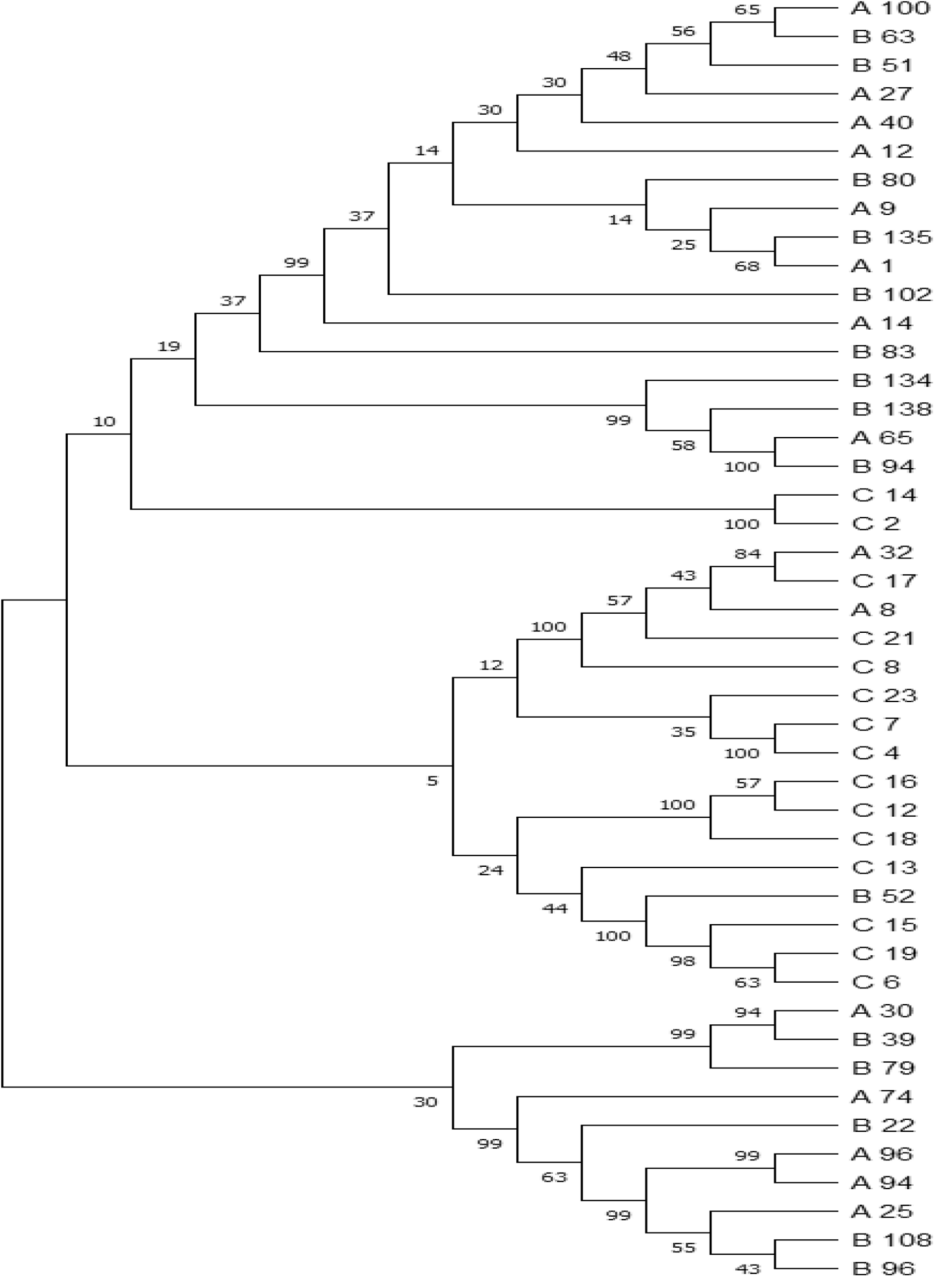
Phylogenetic tree represent the relationship between the *k 13*-propeller genes extracted from the *P.falciparum* parasites isolated from Jazan region in 2011 (A samples), 2016 (B samples) and 2020 (C samples**)**

The phylogenetic tree constructed for 45 samples (15 samples from each time group as example) using the Molecular Evolutionary Genetic Analysis (MEGA 5) represents the evolutionary relationship based upon the genetic characterization between the *k13*-propeller genes extracted from the parasites collected in 2011, 2016 and 2020. It also showed the *k13* genes of 2011 samples genetically more related to the 2016 samples *k13* genes than 2020 samples. In addition the *k13* genes of 2020 samples are closely related to each other (Fig. 4).

## Discussion

One of the most important health directives for ensuring global elimination of malaria is increasing molecular surveillance and monitoring for mutations that would develop parasite resistance to anti-malarial drugs (including ACT). ACT resistance is prevalent in SEA and China [18, 19] while South America, Central and South Asia, Southwest Asia, Africa, Oceania, and the Philippines are free of NS k13 mutations. There was no evidence of invasion into Africa by Asian artemisinin-resistant alleles, while NS mutations were observed with low frequency. Further, these mutations were not associated with clinical ACT resistance [20].

The involvement of K13 mutations in ACT resistance in different countries of Asia necessitates molecular surveillance of *k13* genes in many malaria-endemic regions.

This is the first published study in Saudi Arabia profiling mutations at *k13*-propeller; some of these SNPs may have a future role in ACT resistance in the Jazan region of southwest Saudi Arabia since the introduction of ACT as first-and second-line treatment for malaria in 2004, to prevent artemisinin resistance from becoming established in this area.

The aim of this study was to evaluate the development of ACT resistance in the Jazan region and to track the evolution of the *k13* gene from 2011 to 2020. Six SNPs (11.8%) found only in 2011 samples, 3 SNPs (5.9%) in 2011 and 2016, 5 SNPs (9.8%) in 2011 and 2020, 5 SNPs (9.8%) in 2016 only, 8 SNPs (15.7%) in 2016 and 2020, 14 SNPs (27.5%) in 2020, while 10 SNPs (19.6%) were present in all three time groups. This finding revealed that the mutations in 2020 samples were higher than in 2011 and 2016 samples. Furthermore, mutations in 2020 samples were distributed in all blades, while mutations in 2011 and 2016 samples were distributed in blades 1, 5 and 6. In general, blade 6 contained most of the identified mutations in all the three groups.

Sequencing of *k13* gene from the 140 collected parasite samples in the Jazan region indicated that 7 of these mutations were previously observed in the SEA region, compared to 8 SNPs reported in SSA and 4 in both the regions [12, 21–23]. These mutations did not appear at the same time, except for V666A and D680N (SSA mutations), which were detected in all the three time groups.

Eight mutations from those reported by WHO as validated or associated with ACT resistance appeared at low frequencies, at different times, emerging independently, with most of them in the same codon position albeit with different amino acid substitution, except M476I. It is well known that most proteins can withstand one or two points mutation before their function changes [24].

2020 sampled parasites have the most private polymorphic *k13* gene with a total 14 SNPs, compared to 6 in 2011 samples and 5 in 2016 samples. This finding along with the BLAST analysis showed that Jazan parasites have many polymorphisms at low frequencies and emerged independently in every time group and that the genetic structure for *k13* gene is not stable. This corroborates the previous studies reported that in Africa parasites have a large excess of polymorphisms with minor allele frequencies, which are evenly distributed across the genome [25, 26].

Few studies have been published regarding countries in East and Central Africa that are close to Jazan region. A recent study conducted in Tanzania revealed that 26 NS k13 SNPs were detected in Tanzanian samples, including K13 R561H and K13 A578S haplotypes validated to cause artemisinin resistance and not associated with artemisinin resistance [27]. In Kenya about 19 NS mutations were present in pre-ACT samples compared with 22 in post-ACT samples [26].

An identity and distance tree from BLAST revealed that 2011 isolates were genetically closer to African isolates (53.3%) than Asian ones (46.7%). Interestingly, this proportion changed completely in the 2020 isolates to become closer to Asian isolates, reaching 81.6% (China accession numbers MN586248- MK877456- MK877298- MN586257, India MK949522- KX575553- KY799148- KX575639, Singapore MH341707, Myanmar KM192268), than African ones, which decreased to 18.4% (Nigeria MH464879- MH464877, Kenya MN072988). This finding can be linked to the usage of the drug for a long time, which probably led to drug resistance and fitness in the malaria parasite.

All previous studies in *k13* gene were done in SEA and SSA regions and no studies have been conducted before in Southwest Asia with which to compare results.

This study should have been carried out with in vivo and in vitro assays to discover whether the detected mutations have any effect on ACT resistance. The sample size is not adequate to give comprehensive analytical information about parasite ACT resistance; it is the first study of its type in the region.

## Conclusions

*k13*–propeller polymorphisms spread in different geographical regions where malaria is endemic, but the mutations associated with ACT resistance are exclusive to date to SEA countries. Genomic studies are one of the routine recommendations by the WHO in malaria elimination programmes.

Despite the diversity of identified mutations in the *k13* gene, data did not report the spread of artemisinin-resistant polymorphisms in Jazan region when these samples were collected. However, *k13*-propeller mutations may assist in promoting the evolution of the partner drug resistance [28]. Such a process would be expected to increase the frequencies of mutations associated with ACT resistance. This study indicates baseline prevalence of *k13*-propeller mutations in Jazan region with samples collected in three different times: 2011, 2016 and 2020. This baseline information will be essential in tracking and monitoring of *P. falciparum* resistance to artemisinin in Jazan region.

Correlation of the present results of genetic researches with in vivo and in vitro assays is needed to identify the functional role of detected mutations as markers of artemisinin resistance in Jazan region.

## Data Availability

The data used to support the findings of this study are available from the National Center for Disease Prevention and Control, Saudi Arabia (SCDC).

## Abbreviations

ACT: Artemisinin-based combination therapy
K13: Kelch 13 gene
SEA: Southeast Asia
SSA: Sub-Saharan Africa
DNA: Deoxyribonucleic acid; pfkelch13 gene
BLAST: Basic local alignment search tool
PCR: Polymerase chain reaction
bp: Base pair
NS: Non-synonymous
SNPs: Single nucleotide polymorphisms
WHO: World Health Organization
CQ: Chloroquine
SP: Sulfadoxine pyrimethamine
ssRNA: Small subunit ribosomal RNA genes
